# Biotin concentration affects anaplerotic reactions functioning in glutamic acid production in Corynebacterium glutamicum

**DOI:** 10.1099/mic.0.001507

**Published:** 2024-10-07

**Authors:** Takako Ochiai, Masaaki Wachi, Takashi Hirasawa

**Affiliations:** 1 School of Life Science and Technology, Tokyo Institute of Technology, 4259 Nagatsuta-cho, Midori-ku, Yokohama, Kanagawa 226-8501, Japan

**Keywords:** anaplerotic reaction, biotin, *Corynebacterium glutamicum*, glutamic acid, phosphoenolpyruvate carboxylase, pyruvate carboxylase

## Abstract

The study investigates the effect of biotin concentration on the role of anaplerotic reactions catalysed by pyruvate carboxylase (PC) and phosphoenolpyruvate carboxylase (PEPC) in glutamic acid production by *Corynebacterium glutamicum. C. glutamicum* requires biotin for its growth, and its glutamic acid production can be induced by the addition of Tween 40 or penicillin or by biotin limitation. The biotin enzyme PC and the non-biotin enzyme PEPC catalyse two anaplerotic reactions to supply oxaloacetic acid to the TCA cycle in *C. glutamicum*. Therefore, they are crucial for glutamic acid production in this bacterium. In this study, we investigated the contribution of each anaplerotic reaction to Tween 40- and penicillin-induced glutamic acid production using disruptants of PEPC and PC. In the presence of 20 µg l^–1^ biotin, which is sufficient for growth, the PEPC-catalysed anaplerotic reaction mainly contributed to Tween 40- and penicillin-induced glutamic acid production. However, when increasing biotin concentration 10-fold (i.e. 200 µg l^–1^), both PC- and PEPC-catalysed reactions could function in glutamic acid production. Western blotting revealed that the amount of biotin-bound PC was reduced by the addition of Tween 40 and penicillin in the presence of 20 µg l^–1^. However, these induction treatments did not change the amount of biotin-bound PC in the presence of 200 µg l^–1^ biotin. These results indicate that both anaplerotic reactions are functional during glutamic acid production in *C. glutamicum* and that biotin concentration mainly affects which anaplerotic reactions function during glutamic acid production.

## Introduction

A non-pathogenic coryneform bacterium, *Corynebacterium glutamicum*, was isolated by Japanese researchers as a glutamic acid-overproducing microorganism in 1965 [1]. Since its discovery, this microorganism has been used in the fermentative production of various amino acids, including lysine [2,3], valine [4], threonine [5] as well as glutamic acid. Currently, annual production of glutamic acid exceeds three million tons.

*C. glutamicum* requires an external source of biotin for its growth because it does not have some of the enzymes required for biotin biosynthesis [6,7]. *C. glutamicum* does not produce glutamic acid in the presence of biotin sufficient for growth, but it can produce glutamic acid under biotin-limited conditions [8]. Under biotin-limited conditions, the growth of *C. glutamicum* is suppressed, but consumption of carbon and nitrogen sources continues, and glutamic acid is produced. Addition of Tween 40 [9] and penicillin [10] to culture also induces glutamic acid production in *C. glutamicum*. During glutamic acid production, activity of the 2-oxoglutarate dehydrogenase complex, which catalyses the conversion of 2-oxoglutarate to succinyl-CoA in the TCA cycle, decreases, and the metabolic flow from 2-oxoglutarate to glutamic acid increases [11,12]. Therefore, the metabolic flow from 2-oxoglutarate to oxaloacetate in the TCA cycle is reduced, and consequently the synthesis of oxaloacetate required for citrate synthesis is also reduced. To maintain metabolic flow from oxaloacetate and acetyl-CoA to 2-oxoglutarate in the TCA cycle during glutamic acid production in *C. glutamicum*, oxaloacetate has to be replenished to the TCA cycle by alternative reactions. Anaplerotic reactions replenish oxaloacetate from phosphoenolpyruvate or pyruvate synthesized in the glycolysis.

**Fig. 1.**
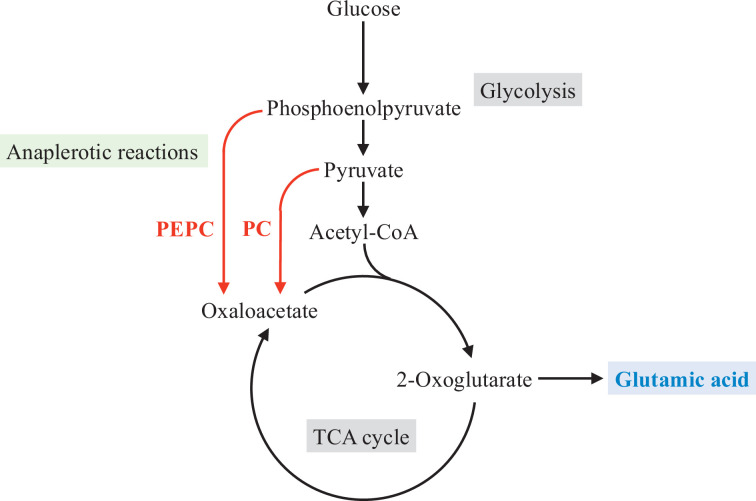
Metabolic pathways of *C. glutamicum* including anaplerotic reactions and glutamic acid production.

*C. glutamicum* has two anaplerotic reactions catalysed by phosphoenolpyruvate carboxylase (PEPC) and pyruvate carboxylase (PC) (Fig. 1). PEPC encoded by the *ppc* gene catalyses the conversion of phosphoenolpyruvate and bicarbonate into oxaloacetate and inorganic phosphate [13,14]. PC encoded by the *pyc* gene catalyses the conversion of pyruvate and bicarbonate to oxaloacetate, which requires ATP hydrolysis [15,16]. Notably, PC contains a biotin prosthetic group, and biotin is required for its activity [15]. Several studies have explored the contribution of anaplerotic reactions to glutamic acid production under various induction conditions in *C. glutamicum*. As expected, PEPC is responsible for glutamic acid production under biotin-limited conditions because PC, which requires biotin for its activity, cannot function under such conditions [17]. Moreover, ^13^C-metabolic flux analysis by Shirai *et al*. [18] revealed that PC is important for Tween 40-induced glutamic acid production. Nagano-Shoji *et al*. [19] found that PEPC is a key anaplerotic pathway enzyme for Tween 40-induced glutamic acid production using *ppc* and *pyc* disruptants. Peters-Wendisch *et al*. [20] demonstrated that PC is a major bottleneck of Tween 60-induced glutamic acid production using *pyc* overexpression and disrupted strains. Enzyme activity measurements conducted during glutamic acid production by Hasegawa *et al*. [21] indicated that PC activity is comparatively higher when Tween 40 and penicillin are added but lower under biotin-limited conditions than normal growth conditions. In addition, PEPC activity under glutamic acid production conditions is lower than under normal growth conditions [21]. Delaunay *et al*. [22] reported the importance of PEPC in temperature-triggered glutamic acid production in *C. glutamicum*.

As described above, there are some inconsistencies in the previous studies regarding the importance of anaplerotic reactions during glutamic acid production by *C. glutamicum* probably because of differences in biotin concentrations, strain background and culture systems (Table S1, available in the online version of this article). Among such factors, it is thought that biotin concentration critically affects the role of anaplerotic reactions in glutamic acid production by *C. glutamicum*, because PC requires biotin for its activity and biotin concentration is related to induction of glutamic acid production. In the present study, we investigated the effect of biotin concentration on Tween 40- and penicillin-induced glutamic acid production by *C. glutamicum* and the contribution of each anaplerotic reaction to glutamic acid production using disruptants of anaplerotic reaction enzymes. We found that PEPC is important for glutamic acid production but PC can function when the concentration of biotin in the culture medium is increased.

## Methods

### Strains and media

*C. glutamicum* wild-type strain ATCC 31831 and its *ppc* and *pyc* disruptants that were constructed in a previous study [17] were used in this study.

Lennox agar medium, CM2B medium and synthetic glutamic acid production medium were used for glutamic acid production assays. Lennox agar medium consists of (per litre) 10 g hipolypepton (Shiotani M.S.), 5 g dried yeast extract D-3H (Shiotani M.S.), 5 g NaCl, 1 g glucose and 15 g agar (pH 7). CM2B medium consists of (per litre) 10 g hipolypepton N (Shiotani M.S.), 10 g dried yeast extract D-3H, 5 g NaCl and 10 g biotin (pH 7.2). Synthetic glutamic acid production medium consists of (per litre) 80 g glucose, 24 g (NH_4_)_2_SO_4_, 3.0 g Na_2_HPO_4_·12H_2_O, 6.0 g KH_2_PO_4_, 2.0 g NaCl, 84 mg CaCl_2_, 3.9 mg FeCl_3_, 0.4 mg ZnCl_2_, 0.3 mg CuCl_2_·2H_2_O, 4.6 mg MnCl_2_·4H_2_O, 0.1 mg (NH_4_)_6_Mo_7_O_24_·4H_2_O, 0.3 mg Na_2_B_4_O_7_·10H_2_O, 0.3 g MgCl_2_·6H_2_O, 29 mg FeCl_2_·4H_2_O, 500 µg thiamine hydrochloride, 0.1 g EDTA, and 10, 20 or 200 µg d-biotin (pH 7.2). To maintain the pH in the culture during the glutamic acid production assay, 25 g l^–1^ CaCO_3_ was added to the synthetic glutamic acid production medium. In addition, soybean hydrolysates containing 0.2 g nitrogen l^–1^ or 10 g l^–1^ dried yeast extract D-3H was added to the synthetic glutamic acid production medium to improve cell growth and glutamic acid production.

### Glutamic acid production assay

A seed culture was prepared by inoculating a single colony of *C. glutamicum* grown on Lennox agar plates into 5 ml of CM2B medium and incubating it at 30 °C for 24 h. The seed culture was then transferred to 40 ml of synthetic glutamic acid production medium containing 10 µg l^–1^ d-biotin in a 300 ml baffled flask and incubated with rotary shaking at 200 r.p.m. at 30 °C for 24 h to make the preculture. For the main culture of the glutamic acid production assay, 1 ml of the preculture was transferred to 40 ml of synthetic glutamic acid production medium containing 20 or 200 µg l^–1^ d-biotin in a baffled flask and incubated with rotary shaking at 200 r.p.m. at 30 °C. To induce glutamic acid production, 4 g l^–1^ Tween 40 or 10 µM penicillin G was added 4 and 6 h after starting cultivation, respectively, when the cell growth reached the mid-exponential phase, at which the optical density of the culture at 660 nm (OD_660_) reached 10–15. During the main culture, samples of cells and culture supernatants were collected by centrifugation for further analysis.

### Measurement of cell growth and concentration of glucose, glutamic acid and lactate

Cell growth was monitored by measuring OD_660_ using a UV-1280 spectrophotometer (Shimadzu). Before measuring OD_660_, cultures were diluted with 0.2 M HCl to dissolve CaCO_3_ in the cultures. The glucose, glutamic acid and lactate concentrations in the culture supernatant were measured using a Glucose CII test Wako (Fujifilm Wako Pure Chemical), F-kit l-glutamate (Roche Diagnostics) and F-kit l-lactate (Roche Diagnostics), respectively.

### Western blotting

Cells suspended in 50 mM sodium phosphate buffer (pH 7.0) were disrupted by sonication, and cell debris was removed by centrifugation to obtain crude extracts of *C. glutamicum* cells. After measuring the protein concentration in crude extracts using protein assay dye reagent concentrate (Bio-Rad Laboratories), the extracts containing 10 µg protein were mixed with sample buffer solution with reducing reagent (6×) for SDS-PAGE (Nacalai Tesque) and then boiled for 3 min. Subsequently, the samples were subjected to SDS-PAGE using a 12.5% acrylamide running gel. Proteins separated in the gel were transferred electrically to a PVDF membrane (Amersham Hybond P PVDF 0.2; Cytiva), and biotinylated proteins, such as PC and acetyl-CoA carboxylase (ACC), on the membrane were detected using a streptavidin-horseradish peroxidase conjugate (Cell Signaling Technology), Amersham ECL prime western blotting detection reagent (Cytiva) and Hyperfilm-ECL (Cytiva).

### Reverse transcription-quantitative PCR (RT-qPCR) analysis of the *pyc* gene

*C. glutamicum* ATCC 31831 was cultured and glutamic acid production was induced by the same procedures as those for glutamic acid production assays. Cells were harvested by centrifugation 6 h after addition of 4 g l^–1^ Tween 40 and 10 µM penicillin G to the cultures and stored at –80 °C until total RNA extraction. Total RNA was extracted by a NucleoSpin RNA (Macherey-Nagel). Reverse transcription of total RNA was performed using ReverTra Ace qPCR RT Master Mix with gDNA Remover (Toyobo). Real time PCR was conducted using a Thermal Cycler Dice Real Time System III (Takara Bio), Thunderbird Next SYBR qPCR Mix (Toyobo) and primer sets 5′-ACCTGGTGAAGGCGCAGATG-3′ and 5′-ACCGTCAAGACGAACGCCAG-3′ for *pyc*, and 5′-CTTACCTGGGCTTGACATGG-3′ and 5′-CACCACAATGTGCTGGCAAC-3′ for the 16S rRNA gene. The primers were designed based on the genome sequence of ATCC 31831 (DDBJ/ENA/GenBank accession numbers BLRJ01000001.1 and BLRJ01000002.1) [23] using Primer-BLAST (https://www.ncbi.nlm.nih.gov/tools/primer-blast/index.cgi). Fold change in expression of the *pyc* gene in the presence of 200 µg l^–1^ biotin compared with that in the presence of 20 µg l^–1^ biotin was calculated by the ∆∆Ct method using Ct value for the 16S rRNA gene as a housekeeping gene.

## Results

### Effect of biotin concentrations on Tween 40-induced glutamic acid production in the anaplerotic reaction disruptants of *C. glutamicum*

As described above, it was thought that biotin concentration is critical for differential utilization of anaplerotic reactions in glutamic acid production by *C. glutamicum*. Therefore, we investigated the effects of changes in biotin concentration on glutamic acid production using the disruptants of the anaplerotic reactions in *C. glutamicum*. In the present study, we used the *ppc* and *pyc* disruptants which were previously constructed from the wild-type strain ATCC 31831 [17] and cultured them in the same medium containing different concentrations of biotin for glutamic acid production. Under biotin-sufficient conditions, only PC can function in the *ppc* disruptant while only PEPC can function in the *pyc* disruptant.

**Fig. 2.**
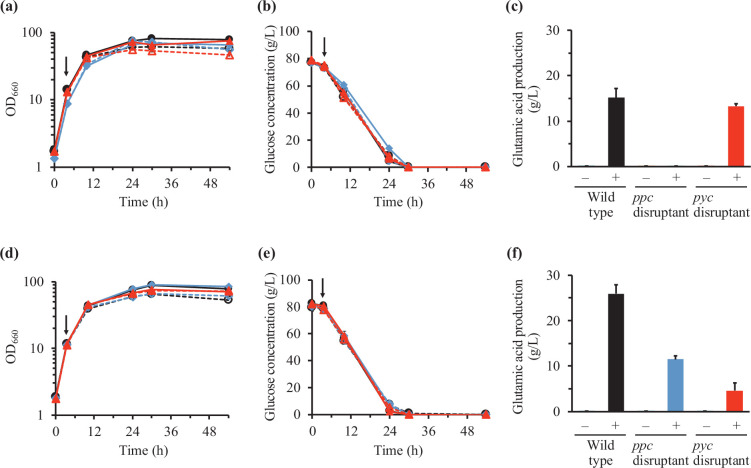
Effect of biotin concentration on Tween 40-induced glutamic acid production in the wild-type strain and *pyc* and *ppc* disruptants of *C. glutamicum*. Time courses of cell growth (**a and d**) and glucose concentration in the culture supernatants (**b and e**) in the wild-type (black circles), *pyc* disruptant (red triangles) and *ppc* disruptant (blue diamonds) without (filled symbols with solid lines) and with (open symbols with dotted lines) Tween 40 addition in the presence of 20 (**a and b**) and 200 (**d and e**) µg l^–1^ biotin are shown. Arrows represent the addition of Tween 40 (4 h after starting cultivation). In addition, glutamic acid production 30 h after starting cultivation without (–) and with (+) Tween 40 addition in the presence of 20 (**c**) and 200 (**f**) µg l^–1^ biotin is shown. Mean±sd from three independent culture experiments is shown.

First, Tween 40-induced glutamic acid production by the *pyc* and *ppc* disruptants in the presence of 20 µg l^–1^ biotin was analysed. Tween 40 (4 g l^–1^) was added to the cultures at the mid-exponential growth phase, 4 h after starting cultivation. As shown in Fig. 2(a), *pyc* and *ppc* disruptants as well as the wild-type strain could grow well, indicating that 20 µg l^–1^ biotin is sufficient for the growth of *C. glutamicum*. Although the addition of Tween 40 slightly reduced cell growth in all three strains (Fig. 2a), most glucose input was consumed 30 h after starting cultivation (26 h after Tween 40 addition) (Fig. 2b). Tween 40 addition induced glutamic acid production in the wild-type and *pyc* disruptant but not in the *ppc* disruptant (Fig. 2c). Furthermore, glutamic acid production levels at 30 h were similar in the wild-type strain and *pyc* disruptant (Fig. 2c). These results indicate that PEPC is important for Tween 40-induced glutamic acid production in *C. glutamicum* in the presence of 20 µg l^–1^ biotin.

Next, the biotin concentration in the medium was increased to 200 µg l^–1^, which is 10-fold the concentration required for the growth of *C. glutamicum*, and glutamic acid production induced by Tween 40 addition in the *pyc* and *ppc* disruptants was analysed. Growth properties in the three strains were similar to those in the presence of 20 µg l^–1^ biotin (Fig. 2a, d). Glucose was completely consumed at 30 h in all strains examined, and glutamic acid production at 30 h was observed in the *pyc* disruptant as well as the wild-type strain (Fig. 2e, f). However, glutamic acid production was reduced in the *pyc* disruptant compared to that in the presence of 20 µg l^–1^ biotin. Interestingly, the *ppc* disruptant produced glutamic acid by Tween 40 addition in the presence of 200 µg l^–1^ biotin as well (Fig. 2f), indicating that both PC- and PEPC-catalysed reactions can function in Tween 40-induced glutamic acid production under increased biotin concentrations.

### Effect of biotin concentrations on penicillin-induced glutamic acid production in the anaplerotic reaction disruptants of *C. glutamicum*

**Fig. 3.**
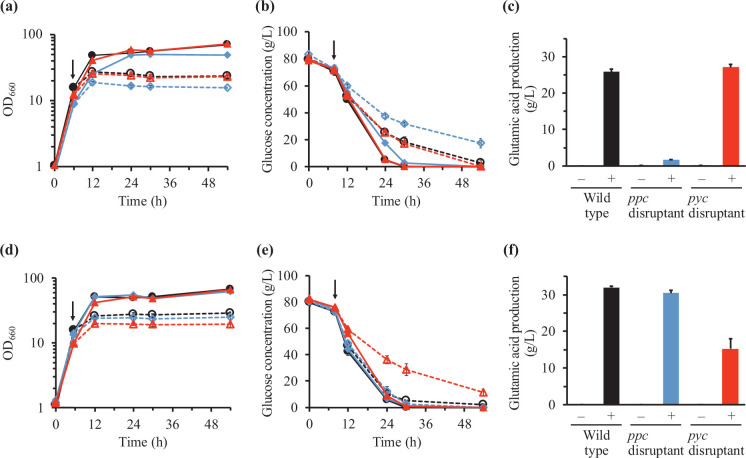
Effect of biotin concentration on penicillin-induced glutamic acid production in the wild-type strain and *pyc* and *ppc* disruptants of *C. glutamicum*. Time courses of cell growth (**a and d**) and glucose concentration in the culture supernatant (**b and e**) in the wild-type (black circles), *pyc* disruptant (red triangles) and *ppc* disruptant (blue diamonds) without (filled symbols with solid lines) and with (open symbols with dotted lines) penicillin addition in the presence of 20 (**a and b**) and 200 (**d and e**) µg l^–1^ biotin are shown. Arrows represent the addition of penicillin (6 h after starting cultivation). In addition, glutamic acid production 54 h after starting cultivation without (–) and with (+) penicillin addition in the presence of 20 (**c**) and 200 (**f**) µg l^–1^ biotin is shown. Mean±sd from three independent culture experiments is shown.

We also investigated the effect of biotin concentration on penicillin-induced glutamic acid production by *C. glutamicum*. Penicillin (10 µM) was added to the cultures at the mid-exponential phase, 6 h after starting cultivation. The addition of penicillin G reduced cell growth of the wild-type strain and anaplerotic reaction disruptants (Fig. 3a). Upon penicillin addition, most glucose was consumed at 54 h (i.e. 48 h after penicillin addition) in the wild-type strain and *pyc* disruptant, but one-fifth of the glucose input remained in the *ppc* disruptant 54 h after starting cultivation (Fig. 3b). All three strains produced glutamic acid after penicillin addition 54 h after starting cultivation (Fig. 3c). However, glutamic acid production levels were negligible in the *ppc* disruptant compared with those in the wild-type strain and *pyc* disruptant (Fig. 3c). These results indicate that, in the presence of 20 µg l^–1^ biotin, PEPC is significant for penicillin-induced glutamic acid in *C. glutamicum*.

In the presence of 200 µg l^–1^ biotin, the addition of penicillin halted cell growth, and almost all glucose input was consumed at 30 h (i.e. 24 h after penicillin addition) in the wild-type strain and *ppc* disruptant (Fig. 3d, e). In contrast, glucose consumption was lower in the *pyc* disruptant than in the wild-type strain and *ppc* disruptant, and one-eighth of the glucose input remained at 54 h. Glutamic acid production levels at 54 h in the *ppc* disruptant were similar to those in the wild-type strain (Fig. 3f). However, they were lower in the *pyc* disruptant (Fig. 3f). These outcomes differ from those in the presence of 20 µg l^–1^ biotin, indicating that both PC and PEPC can contribute to penicillin-induced glutamic acid production in the presence of 200 µg l^–1^ biotin.

### Detection of biotin binding to PC during glutamic acid production by Western blotting

Biotin binds PC and is required for its activity [15]. It was speculated that the binding property of biotin to PC is altered by changing the biotin concentrations in the culture medium, and that such alterations in biotin binding are reflected in the properties of glutamic acid production. Therefore, we analysed biotin binding to PC during glutamic acid production by Western blotting using streptavidin, a biotin-binding protein.

**Fig. 4.**
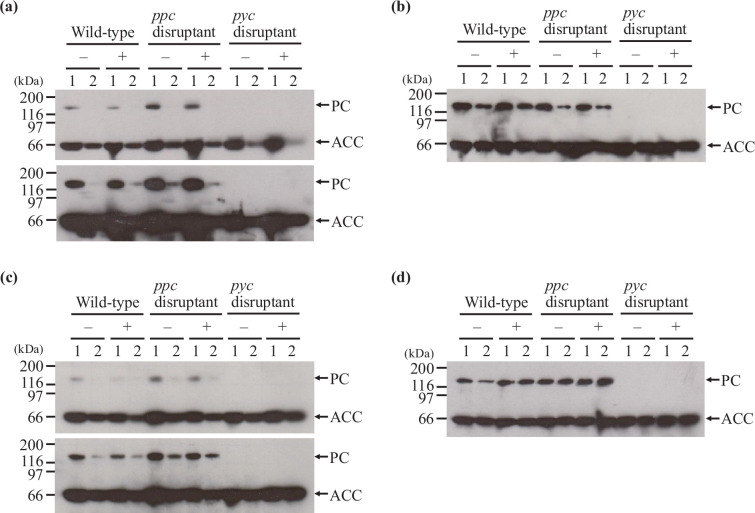
Detection of biotin binding to pyruvate carboxylase in glutamic acid production. Biotin-bound pyruvate carboxylase (PC) and acetyl-CoA carboxylase (ACC) without (–) and with (+) Tween 40 (**a and b**) and penicillin (**c and d**) addition in the presence of 20 (**a and c**) and 200 (**b and d**) µg l^–1^ biotin was detected by Western blotting. In the case of Tween 40 addition, lanes 1 and 2 represent samples obtained at 4 and 10 h after starting cultivation, which correspond to immediately before and 6 h after Tween 40 addition, respectively. In the case of Tween 40 addition, lanes 1 and 2 represent samples obtained at 6 and 12 h after starting cultivation, which correspond to immediately before and 6 h after penicillin addition, respectively. Lanes 1 and 2 without Tween 40 and penicillin addition represent samples obtained at the same time as those obtained with Tween 40 and penicillin addition (i.e. 4 and 10 h and 6 and 12 h starting cultivation without Tween 40 and penicillin addition, respectively). The lower images in (a) and (c) are exposed for longer than the upper images. Numbers on the left of the images represent the molecular mass (kDa).

In this experiment, Tween 40 was added to the cultures 4 h after starting cultivation and the cells were harvested immediately before and 6 h after Tween 40 addition to prepare crude extracts for Western blotting. As shown in Fig. 4(a), biotin-bound PC was not detected 6 h after Tween 40 addition in the wild-type strain or *ppc* disruptant. However, biotin-bound PC was detected following Tween 40 addition after a longer film exposure (Fig. 4a, lower panel), revealing that its amount was lower than that before addition. This result suggests that biotin-bound PC levels in the presence of 20 µg l^–1^ biotin seem to be insufficient for the *ppc* disruptant to produce glutamic acid by Tween 40 addition.

Compared to the result in the presence of 20 µg l^–1^ biotin, biotin-bound PC was clearly detected before Tween 40 addition and even at 6 h after Tween 40 addition in both the wild-type strain and *ppc* disruptant, and its amount was not reduced by adding Tween 40 in the presence of 200 µg l^–1^ biotin (Fig. 4b). This was consistent with the glutamic acid production by the *ppc* disruptant upon Tween 40 addition in the presence of 200 µg l^–1^ biotin.

Furthermore, we analysed the effect of biotin concentration on binding of biotin to PC upon penicillin addition. Penicillin G was added to the cultures 6 h after starting cultivation and the cells were harvested just before and 6 h after penicillin addition to prepare crude extracts for Western blotting. As depicted in Fig. 4(c), in the presence of 20 µg l^–1^ biotin, the amount of biotin-bound PC decreased in the wild-type strain and *ppc* disruptant 6 h after the addition of penicillin compared to that before addition. It is thought that this phenomenon causes a reduction in penicillin-induced glutamic acid production in the *ppc* disruptant (Fig. 4c). As observed with the addition of Tween 40, in the presence of 200 µg l^–1^ biotin, biotin binding to PC was not reduced 6 h after addition of penicillin in the wild-type strain and *ppc* disruptant (Fig. 4d). This result indicates that biotin-bound PC can contribute to penicillin-induced glutamic acid production in the presence of 200 µg l^–1^ biotin. As expected, biotin-bound PC was not detected in the *pyc* disruptant (Fig. 4).

### Effect of biotin concentration on expression of the *pyc* gene in *C. glutamicum*

The amount of biotin-bound PC was decreased after inducing glutamic acid production with Tween 40 and penicillin (Fig. 4). One possible reason for this phenomenon is that the total amount of PC (i.e. biotin-bound PC plus biotin-free PC) in the presence of 200 µg l^–1^ biotin might become larger than that in the presence of 20 µg l^–1^ biotin after inducing glutamic acid production. However, Western blotting using streptavidin cannot determine the total amount of PC because streptavidin can detect only biotin-bound PC. In addition, it has not been reported yet whether biotin concentration affects expression of the *pyc* gene encoding PC or not. Therefore, we analysed expression levels of the *pyc* gene in the presence of 200 µg l^–1^ biotin compared with those in the presence of 20 µg l^–1^ biotin in the wild-type ATCC 31831 strain after inducing glutamic acid production. For total RNA extraction, the ATCC 31831 strain was cultured by the same procedures as those for the glutamic acid production assay; Tween 40 and penicillin were added 4 and 6 h after starting cultivation, respectively. Here, the difference in expression levels of *pyc* 6 h after adding Tween 40 and penicillin in strain ATCC 31831 in the presence of 20 and 200 µg l^–1^ biotin was analysed.

**Fig. 5.**
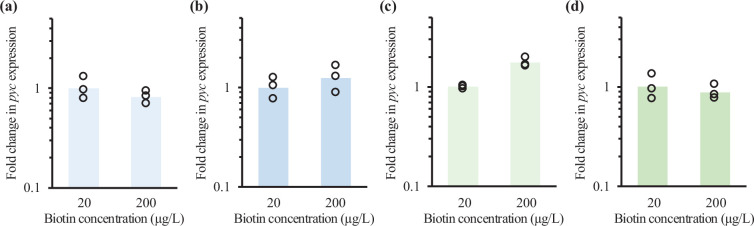
Effect of biotin concentration on expression of the *pyc* gene after inducing glutamic acid production. Fold change in expression of *pyc* gene in the presence of 200 µg l^–1^ biotin relative to that in the presence of 20 µg l^–1^ biotin in the wild-type strain ATCC 31831 10 h after starting cultivation without Tween 40 addition (**a**) and with Tween 40 addition (i.e. 6 h after Tween 40 addition) (**b**) is shown. Moreover, fold change in expression of the *pyc* gene in the presence of 200 µg l^–1^ biotin compared with that in the presence of 20 µg l^–1^ biotin in strain ATCC 31831 12 h after starting cultivation without penicillin addition (**c**) and with penicillin addition (i.e. 6 h after penicillin addition) (**d**) is shown. Fold change in *pyc* gene expression was calculated by the ΔΔCt method using the Ct value for the 16S rRNA gene as a housekeeping gene. Circles and bars represent individual fold changes in the cells from three independent cultures and their geometric mean, respectively.

Under the culture conditions with and without Tween 40 addition, the difference in *pyc* expression levels between in the presence of 20 and 200 µg l^–1^ biotin was not observed (Fig. 5a, b), indicating that biotin concentration does not affect *pyc* gene expression after inducing glutamic acid production by Tween 40 addition. In addition, expression of the *pyc* gene in the presence of 200 µg l^–1^ biotin was higher than that in the presence of 20 µg l^–1^ biotin under the culture conditions without penicillin addition, but the difference was small (Fig. 5c). The difference in *pyc* expression levels between in the presence of 20 and 200 µg l^–1^ biotin after inducing glutamic acid production by penicillin addition was not observed (Fig. 5d), indicating that biotin concentration does not affect *pyc* gene expression after inducing glutamic acid production by penicillin addition as well. Considering the results of *pyc* gene expression analysis, it is thought that the total amount of PC in the presence of 200 µg l^–1^ biotin is similar to that in the presence of 20 µg l^–1^ biotin, but the amount of biotin-bound PC is changed by biotin concentration.

## Discussion

In this study, we investigated the effect of biotin concentration on the contribution of anaplerotic reactions to glutamic acid production in *C. glutamicum*. We found that both PC and PEPC can function in glutamic acid production in *C. glutamicum* and that the biotin concentration affected the role of the anaplerotic reactions. The *ppc* disruptant could not produce glutamic acid upon Tween 40 addition and produced negligible amounts upon penicillin addition in the presence of 20 µg l^–1^ biotin, which is sufficient for the growth of *C. glutamicum* (Figs2c  3c), indicating that the PEPC-catalysed reaction dominates in glutamic acid production by *C. glutamicum*. This was consistent with the results of Western blotting to detect biotin-bound PC; the amount of biotin-bound PC was reduced by the addition of Tween 40 and penicillin in the presence of 20 µg l^–1^ biotin (Fig 4a, c). Similar results were reported in the literature [24,25]; biotin concentration affects intracellular levels of biotin-bound PC in *C. glutamicum* grown on lactate.

It could be speculated that the decrease in the levels of biotin-bound PC after inducing glutamic acid production in the presence of 20 µg l^–1^ biotin compared with those in the presence of 200 µg l^–1^ was caused by the decrease in total amount of biotin-bound PC plus biotin-free PC. However, the total amount of biotin-bound PC plus biotin-free PC cannot be determined by Western blotting using streptavidin because only biotin-bound PC can be detected by streptavidin. In addition, in the previous reports, biotin affects expression of the genes related to biotin metabolism in *C. glutamicum*. Schneider *et al*. reported that biotin affected expression of the *bioYMN* operon encoding biotin uptake system [26]. Brune *et al*. reported that expression of the *bioYMN* operon and the genes encoding biotin biosynthesis enzymes was regulated by the transcriptional regulator BioQ, which binds to upstream regions of these genes on the *C. glutamicum* genome [27]. At present, it has not yet been reported whether biotin concentration affects expression of the *pyc* gene encoding PC or not. Therefore, we compared expression levels of the *pyc* PC after inducing glutamic acid production in the presence of 200 µg l^–1^ biotin with those in the presence of 20 µg l^–1^ biotin in the wild-type strain ATCC 31831 (Fig. 5). The difference in expression of the *pyc* gene after Tween 40 and penicillin addition between in the presence of 20 and 200 µg l^–1^ biotin was small, assuming that the total level of PC is unchanged by biotin concentration. Therefore, it is thought that biotin concentration may affect whether biotin can bind to PC or not and, as a result, the levels of biotin-bound PC become higher in the presence of 200 µg l^–1^ biotin than in the presence of 20 µg l^–1^ biotin. The increase in the amount of biotin-bound PC might be related to the function of the biotin protein ligase BirA [25], which catalyses attachment of biotin to proteins including PC. Under high biotin concentration conditions, BirA activity to attach biotin to PC would increase and, as a result, the amount of biotin-bound PC is increased.

In the presence of 200 µg l^–1^ biotin, glutamic acid production levels in the *ppc* disruptant was about half of those in the wild-type strain upon Tween 40 addition (Fig. 2d), while glutamic acid production levels in the wild-type strain and *ppc* disruptant were similar upon penicillin addition (Fig. 3d). The mechanism of this phenomenon caused by differences in treatments to induce glutamic acid production is not yet clear, but the difference in biotin-bound PC levels between treatments for inducing glutamic acid production may affect glutamic acid production levels (Fig. 4b, d). Biotin-bound PC was detected after Tween 40 and penicillin addition. However, levels of biotin-bound PC after Tween 40 addition appeared to be reduced while those after penicillin addition were not (Fig. 4b, d). In addition, the difference in induction treatment for glutamic acid production may affect post-translational regulation of PC activity such as acetylation and succinylation of proteins [19,28]. Analysis of post-translational regulation of PC will be effective to understand the mechanisms of glutamic acid production in *C. glutamicum*.

Moreover, in the presence of 200 µg l^–1^ biotin, glutamic acid production by the *pyc* disruptant was lower than that in the presence of 20 µg l^–1^ biotin (Figs2  3). A previous study by Sato *et al*. [17] showed that lactate production was enhanced by *pyc* gene disruption under biotin-limited conditions, assuming that *pyc* gene disruption results in an increased pool of pyruvate, which is converted to lactate by lactate dehydrogenase. In the present study, high lactate production by the *pyc* disruptant in the presence of 200 µg l^–1^ biotin was observed with and without Tween 40 addition [13.9±3.1 and 17.4±0.9 g l^–1^, respectively, 30 h after starting cultivation (i.e. 26 h after Tween 40 addition)]. The mechanism of increase in lactate production by an increase in biotin concentration is not yet clear, but high lactate production may result in decreased glutamic acid production by the *pyc* disruptant in the presence of 200 µg l^–1^ biotin.

Considering the results in the present study, it is thought that PEPC is utilized in glutamic acid production by *C. glutamicum* but PC can function in the presence of high concentrations of biotin because of an increase in biotin-bound PC levels. The significance of differential utilization of PEPC and PC in cellular metabolism of *C. glutamicum* is not yet fully understood. It has been reported that PEPC and PC activities are regulated by some metabolites; for example, both PEPC and PC activities are inhibited by aspartic acid, and PEPC activity is activated by acetyl-CoA [14,29,31]. The mechanism of differential utilization of PEPC and PC in *C. glutamicum* may contribute to maintenance of balance of intracellular metabolite pools around the phosphoenolpyruvate–pyruvate–oxaloacetate node [32] not only for cell growth but also for glutamic acid production by regulation of PC and PEPC activities with metabolites and probably biotin concentration. Further investigation regarding differential utilization of PEPC and PC in *C. glutamicum* will be necessary.

In addition, metabolic fluxes in anaplerotic reactions are important for lysine production as well as glutamic acid production in *C. glutamicum*. For example, the overexpression or disruption of *ppc* does not affect lysine production [14,33, 34], whereas the overexpression of *pyc* increases lysine production in *C. glutamicum* [20]. Furthermore, deregulation of PEPC against inhibition by aspartic acid and malate improved lysine production by *C. glutamicum* [35]. Recently, analysis of the contribution of anapletoric reactions in lysine production by *C. glutamicum* using *pyc* and *ppc* disruptants was reported [36]. In this study, biotin-bound PC was detected in the PEPC-disrupted lysine-producing strain but reduced lysine production in this strain compared with that in the parental lysine-producing strain; this phenomenon was different from that in our present study. The difference in contribution of biotin-bound PC might be related to the target amino acids produced. Therefore, the relationship between anaplerotic reactions and biotin concentrations need to be analysed to understand the metabolism of production of not only glutamic acid but also other compounds including lysine, whose synthesis is related to oxaloacetate.

## supplementary material

10.1099/mic.0.001507 Uncited Table S1.

## Abbreviations

ACC: acetyl-CoA carboxylase
PC: pyruvate carboxylase
PEPC: phosphoenolpyruvate carboxylase
