# Supplementary material for: Biotin concentration affects anaplerotic reactions functioning in glutamic acid production in Corynebacterium glutamicum
Source: Microbiology (Reading). 2024 Oct 7;170(10):001507. doi: 10.1099/mic.0.001507 (PMC11457263; doi:10.1099/mic.0.001507)
Supplement: Uncited Table S1. [file mic-170-01507-s001.pdf]

Table S1 Summary of studies on contribution of anaplerotic reactions to glutamic acid production in *C. glutamicum*

Induction treatment for glutamic acid production	Method	Biotin concentration in the medium used (μg/L)	Conclusion of which anaplerotic reaction is important for glutamic acid production	Reference
Tween 60 addition	Batch cultivation of the *pyc*-overexpressing strain, *ppc*-overexpressing strain, *pyc* disruptant and *ppc* disruptant, all of which were constructed from the ATCC 13032	2000	PC	[1]
Upshift of temperature from 33°C to 39°C	Batch and fed-batch cultivation of the 2262 strain and its PEPC-amplified strain under biotin sufficient and limitation conditions	4000 (biotin sufficient) or 4 (biotin limitation)	PEPC	[2]
Tween 40 addition	^{13}C metabolic flux analysis of the wild-type ATCC 13869 strain	10	PC	[3]
Biotin limitation, Tween 40 addition and penicillin addition	Measurement of enzyme activity during glutamic acid production in the wild-type ATCC 13869 strain	200 (Tween 40 addition and penicillin addition), 3 (biotin limitation)	PEPC (biotin limitation) or PC (Tween 40 addition and penicillin addition)	[4]
Biotin limitation	Cultivation of the wild-type ATCC 31831 and its *pyc* disruptant and *ppc* disruptant with ^{13}C-glucose and measurement of ^{13}C enrichment in glutamic acid	No addition	PEPC	[5]
Biotin limitation	Batch cultivation of the ATCC 13032 and its mutants harboring PEPC desensitized to feedback inhibition by aspartic acid	3	PEPC	[6]
Combination of biotin limitation and temperature upshift at 0.5°C/5 h from 34°C	Batch and fed-batch cultivation of the GDK-9 strain and its *pyc*-overexpressing strain	1	PC	[7]
Upshift of temperature from 34°C to 39°C	Batch and fed-batch cultivation of the CN1021 strain and its *pyc*-overexpressing strain	600	PC	[7]
Tween 40 addition	Cultivation of the wild-type ATCC 13032 and its *pyc* disruptant and *ppc* disruptant	30	PEPC	[8]

References

1. **Peters-Wendisch PG, Schiel B, Wendisch VF, Katsoulidis E, Mockel B, Sahm H, Eikmanns BJ.** Pyruvate carboxylase is a major bottleneck for glutamate and lysine production by *Corynebacterium glutamicum*. *J Mol Microbiol Biotechnol* 2001;3:295-300
2. **Delaunay S, Uy D, Baucher MF, Engasser JM, Guyonvarch A, Goergen JL.** Importance of phosphoenolpyruvate carboxylase of *Corynebacterium glutamicum* during the temperature triggered glutamic acid fermentation. *Metab Eng* 1999;1:334-343. DOI:10.1006/mben.1999.0131
3. **Shirai T, Fujimura K, Furusawa C, Nagahisa K, Shioya S, Shimizu H.** Study on roles of anaplerotic pathways in glutamate overproduction of *Corynebacterium glutamicum* by metabolic flux analysis. *Microb Cell Fact* 2007;6:19. DOI:10.1186/1475-2859-6-19
4. **Hasegawa T, Hashimoto K, Kawasaki H, Nakamatsu T.** Changes in enzyme activities at the pyruvate node in glutamate-overproducing *Corynebacterium glutamicum*. *J Biosci Bioeng* 2008;105:12-19. DOI:10.1263/jbb.105.12
5. **Sato H, Orishimo K, Shirai T, Hirasawa T, Nagahisa K, Shimizu H, Wachi M.** Distinct roles of two anaplerotic pathways in glutamate production induced by biotin limitation in *Corynebacterium glutamicum*. *J Biosci Bioeng* 2008;106:51-58. DOI:10.1263/jbb.106.51
6. **Wada M, Sawada K, Ogura K, Shimono Y, Hagiwara T, Sugimoto M, Onuki A, Yokota A.** Effects of phosphoenolpyruvate carboxylase desensitization on glutamic acid production in *Corynebacterium glutamicum* ATCC 13032. *J Biosci Bioeng* 2016;121:172-177. DOI:10.1016/j.jbiosc.2015.06.008
7. **Guo X, Wang J, Xie X, Xu Q, Zhang C, Chen N.** Enhancing the supply of oxaloacetate for L-glutamate production by *pyc* overexpression in different *Corynebacterium glutamicum*. *Biotechnol Lett* 2013;35:943-950. DOI:10.1007/s10529-013-1241-3
8. **Nagano-Shoji M, Hamamoto Y, Mizuno Y, Yamada A, Kikuchi M, Shirouzu M, Umehara T, Yoshida M, Nishiyama M, Kosono S.** Characterization of lysine acetylation of a phosphoenolpyruvate carboxylase involved in glutamate overproduction in *Corynebacterium glutamicum*. *Mol Microbiol* 2017;104:677-689. DOI:10.1111/mmi.13658
